# Construction, Interfacial Characteristics, and Stability of External Gelation Soy Protein Isolate–Dextran/Sodium Alginate Water-in-Oil-in-Water Emulsions and Freeze-Dried Microcapsules Loaded with Beech Mushroom-Derived Spermidine

**DOI:** 10.3390/foods15101734

**Published:** 2026-05-14

**Authors:** Chongshu Xia, Min Zhang

**Affiliations:** 1 State Key Laboratory of Food Science and Resources, School of Food Science and Technology, Jiangnan University, Wuxi 214122, China; 2 Jiangsu Province International Joint Laboratory on Fresh Food Smart Processing and Quality Monitoring, Jiangnan University, Wuxi 214122, China; 3 China General Chamber of Commerce Key Laboratory on Fresh Food Processing & Preservation, Jiangnan University, Wuxi 214122, China

**Keywords:** beech mushroom, spermidine, W/O/W double emulsion, external gelation, microcapsules

## Abstract

During thermal processing of the beech mushroom, water-soluble bitterness-related compounds migrate into the cooking liquor. Spermidine (SPD), one of the representative hydrophilic polyamines, has potential nutritional value, but its direct exposure may also contribute to bitterness. To improve its utilization while limiting the direct exposure of SPD, SPD recovered from beech mushroom cooking liquor was used as the core material to prepare soy protein isolate–dextran (SPI–Dex)/sodium alginate (SA) external gelation water-in-oil-in-water (W/O/W) emulsion and freeze-dried microcapsules. The study evaluated SPD recovery, emulsion stability, and the structural and encapsulation properties of the resulting microcapsules. The initial SPD concentration in the cooking liquor was 69.17 mg/L and increased to 520.10 mg/L after membrane filtration, low-temperature concentration, and food-grade enrichment, with an overall recovery of 72.16%. The emulsions showed a typical W/O/W multiple structure, with encapsulation efficiency (EE) and retention efficiency (RE) of 92.90–99.76% and 92.47–96.87%, respectively. SA improved emulsion structure, interfacial charge, and physical stability. After freeze-drying, the microcapsules showed a porous network structure, low water activity (0.2139–0.2279), and low moisture content (2.14–2.88%), with EE of 56.44–98.13% and RE of 70.21–89.12%. These results show that the SPI–Dex/SA system can effectively encapsulate and stabilize beech mushroom-derived SPD, and may provide a feasible strategy for limiting its direct exposure in food systems while improving the utilization of thermal processing by-products.

## 1. Introduction

Beech mushroom (*Hypsizygus marmoreus*) is widely consumed because of its crisp texture, delicate flavor, and high nutritional value. However, thermal processing, particularly steaming and boiling, is often accompanied by an increase in bitterness, thereby impairing overall edible quality and consumer acceptance. Previous studies have shown that the formation of bitterness in processed beech mushrooms does not arise from a single compound, but is associated with the release, migration, and accumulation of multiple water-soluble bitterness-related substances [1]. During thermal processing, these bitterness-related components are transferred from the fruiting body tissues into the aqueous phase, making the cooking liquor an important reservoir for the enrichment of bitter compounds [2].

Spermidine (SPD) is a ubiquitous natural polyamine found in fungi, plants, and animals, and is also one of the representative water-soluble compounds in the beech mushroom. SPD participates in multiple physiological activities, including cell proliferation, autophagy regulation, anti-aging, and the maintenance of intestinal homeostasis, and is therefore considered to possess substantial nutritional and potential functional value [3]. The content and occurrence state of polyamines in foods are influenced by raw material type and processing conditions, while thermal treatment and migration into the aqueous phase may alter their distribution within food systems [4]. Therefore, recovering spermidine from beech mushroom cooking liquor not only represents a means of valorizing bitterness-related compounds that migrate into processing by-products during thermal treatment, but also provides an opportunity to convert substances originally associated with undesirable flavor into reusable functional ingredients.

However, despite its potential functional value, spermidine is a small-molecule, highly hydrophilic polyamine that readily dissolves and diffuses in food systems and may directly contribute to bitterness when exposed in the food matrix. If the recovered SPD is added directly to, or exposed within, a food matrix, it may further intensify bitter stimulation, thereby limiting its feasibility as a functional ingredient [5]. A key challenge in the high-value utilization of SPD lies in retaining its functional benefits while limiting its direct exposure in food systems.

Microencapsulation offers a feasible solution to this challenge. By means of encapsulation, SPD can be confined within a specific carrier structure, thereby reducing its rapid exposure during processing, storage, and the early stages of consumption, as well as limiting its direct contact with the gustatory system. For hydrophilic small-molecule bioactives, W/O/W double emulsions are considered effective delivery systems because the target compounds can be entrapped within the internal aqueous phase, while the oil layer and external aqueous phase provide multiple diffusion barriers [6]. However, W/O/W double emulsions are thermodynamically unstable and vulnerable to phase separation, core leakage, and inner droplet coalescence, all of which have a direct impact on SPD retention and its premature exposure in the external phase [7].

To improve interfacial stability and core retention in double emulsion systems, protein–polysaccharide composite wall materials and external-phase gelation strategies have attracted considerable attention. Following complexation, plant proteins and polysaccharides can form a thickened and more viscoelastic interfacial film at the droplet surface, thereby enhancing emulsion stability through steric hindrance and electrostatic interactions [8]. Sodium alginate, an anionic polysaccharide, can raise the viscosity of the outer aqueous phase and reinforce the continuous-phase network through external-phase structuring or gel formation, thereby limiting droplet migration and core diffusion. Previous research has demonstrated that gelation in the outer water phase enhances W/O/W emulsions’ encapsulation efficacy and physical stability [9]. Compared with SPI-only or Dex-only systems, the SPI–Dex/SA system was designed to provide better emulsion stabilization. SPI can adsorb at the oil–water interface and promote emulsion formation, but protein-only systems may show limited physical stability under processing conditions. Dextran is highly hydrophilic, and polysaccharides generally have weak emulsifying activity, so Dex-only systems are not suitable as sole emulsifier systems. Protein–polysaccharide systems can combine protein adsorption with polysaccharide-derived steric protection, which helps form a thicker interfacial layer and improve emulsion stability [10]. In addition, SPI–dextran systems have been reported to improve the stability of protein-based emulsions compared with SPI-based systems. Therefore, the SPI–Dex/SA formulation was considered more suitable than SPI-only or Dex-only systems for constructing a stable W/O/W delivery structure [11].

On this basis, the present study employed SPD recovered from beech mushroom cooking liquor to develop a SPI–Dex/sodium alginate external gelation W/O/W emulsion and freeze-dried microcapsule system, with the aim of achieving both the valorization of bitterness-related compounds from beech mushroom and the controlled delivery of SPD. The study focused on three aspects: first, the recovery and enrichment of SPD from beech mushroom cooking liquor; second, the enhancement of interfacial stability and encapsulation retention by establishing an external gelation W/O/W delivery system; and third, the systematic evaluation of particle size, zeta potential, rheological behavior, encapsulation performance, storage stability, and structural characteristics at both the emulsion and microcapsule levels, so as to elucidate the application potential of this system in reducing SPD-associated bitter exposure and promoting value-added utilization of bitterness-related compounds.

## 2. Materials and Methods

### 2.1. Materials and Reagents

Fresh beech mushrooms (*Hypsizygus marmoreus*) were obtained from a supermarket in Wuxi, China. Polyglycerol polyricinoleate (PGPR) was obtained from Shanghai Meryer Chemical Technology Co., Ltd. (Shanghai, China). Spermidine, sodium alginate, and sodium chloride were obtained from Macklin Biochemical Co., Ltd. (Shanghai, China). Soy protein isolate (SPI) and dextran 200 were sourced from Shanghai Titan Scientific Co., Ltd. (Shanghai, China) and Suzhou Great Pharmaceutical Technology Co., Ltd. (Suzhou, China), respectively. Soybean oil was purchased commercially in China from Yihai Kerry Arawana Holdings Co., Ltd. (Shanghai, China). HPLC-grade methanol and acetonitrile were supplied by Sinopharm Chemical Reagent Co., Ltd. (Shanghai, China). Unless otherwise indicated, every additional reagent was of analytical grade. Unless otherwise specified, deionized water (1.0 MΩ·cm at 25 °C) was used throughout the experiments, whereas ultrapure water (18.2 MΩ·cm at 25 °C) was used for HPLC-related analyses.

### 2.2. Preparation of SPD-Enriched Beech Mushroom Cooking Liquor

Spermidine (SPD) was recovered and enriched from beech mushroom cooking liquor using a food-grade protocol modified from D’Amen et al. [12]. Briefly, fresh beech mushrooms (20 g) were boiled in 100 g of water for 4 min, and the cooking liquor was collected and filtered through a 0.22 μm aqueous membrane (Nantong Haizhixing Experimental Equipment Co., Ltd., Nantong, China). The filtrate was concentrated under reduced pressure using an R-205 rotary evaporator (Shanghai Shenshun Biotechnology Co., Ltd., Shanghai, China) at below 40 °C until the volume was reduced to 20% of its original volume, and then centrifuged at 8000 r/min (6796× *g*) for 20 min at 4 °C using an H1850 centrifuge (Hunan Xiangyi Laboratory Instrument Development Co., Ltd., Changsha, China) to remove insoluble materials. The supernatant was collected and used as the intermediate concentrate for subsequent enrichment. This concentrate was adjusted to pH > 10.5 (10.8 ± 0.2) using food-grade calcium hydroxide and kept at room temperature for 30 min. Subsequently, 95% food-grade ethanol was added to achieve a final ethanol concentration of 80.0% ± 5% (*v*/*v*). After stirring for 30 min, the mixture was held at 4 °C for 1 h and centrifuged at 10,000 r/min (10,619× *g*) for 20 min at 4 °C using the same centrifuge to remove precipitated impurities. Ethanol was then removed using the same rotary evaporator at 40 °C until near dryness, and the residue was redissolved in deionized water (1.0 MΩ·cm at 25 °C). The solution was further adjusted to pH 2.8 ± 0.2 using food-grade phosphoric acid and allowed to stand for 30 min to obtain the final SPD-enriched fraction. Finally, the solution was reconcentrated, redissolved in deionized water, and filtered through a 0.22 μm membrane to obtain the final SPD-enriched solution used as the internal aqueous phase for encapsulation.

### 2.3. HPLC Determination of Beech Mushroom-Derived Spermidine

Based on the previously established high-performance liquid chromatography (HPLC) procedure and sample pretreatment protocol, the analytical conditions for spermidine (SPD) determination were further adjusted to achieve improved separation and detection performance. SPD content was determined by HPLC using a Shimadzu 1700 system (Shimadzu, Kyoto, Japan) equipped with an LC–20AD pump and an SPD–M20A photodiode array detector. A VertiSep^TM^ GES C18 column (150 mm × 4.6 mm, 5 μm; Vertical Chromatography Co., Ltd., Bangkok, Thailand) was used at 40 °C with water (18.2 MΩ·cm at 25 °C) as mobile phase A and acetonitrile as mobile phase B. The flow rate, injection volume, and run time were 1.0 mL/min, 100 μL, and 30 min, respectively. The gradient program was 95% A/5% B (0–5 min), 70% A/30% B (5–15 min), 50% A/50% B (15–25 min), and 10% A/90% B (25–30 min). Detection was performed at 210 nm. The HPLC method was validated using SPD standard solutions. The linear range was 1–1000 mg/L. The SPD concentration in the original sample was calculated according to the following equation:

(1) $$C=7.9563\times 10^{-6}A$$

where *C* is the SPD concentration in the original sample (mg/L), *A* is the peak area, and 100 is the dilution factor. The calibration showed good linearity, with *R*^2^ = 0.9937. The LOD and LOQ were calculated using the residual standard deviation method. The equations were LOD = 3.3σ/S and LOQ = 10σ/S, where σ is the residual standard deviation, and S is the slope of the calibration curve. The LOD and LOQ were 0.13 mg/L and 0.40 mg/L, respectively.

The total SPD content, recovery, and enrichment factor during the recovery and enrichment process were calculated from the measured SPD concentration and sample volume. The total SPD content was calculated as follows:

(2) $$M=\frac{C\times V}{1000}$$

where *M* is the total SPD content (mg), *C* is the SPD concentration (mg/L), and *V* is the sample volume (mL). Recovery was calculated as follows:

(3) $$Recovery(\%)=\frac{M_{i}}{M_{0}}\times 100$$

where *M_i_* is the total SPD content at each processing stage, and *M*_0_ is the initial total SPD content. The enrichment factor was calculated as follows:

(4) $$Enrichment\;factor=\frac{C_{i}}{C_{0}}\times 100$$

where *C_i_* is the SPD concentration at each processing stage, and *C*_0_ is the initial SPD concentration.

### 2.4. Preparation of External Gelation W/O/W Emulsions

SPD recovered and enriched from beech mushroom cooking liquor, as described in Section 2.2, was used as the core material to prepare soy protein isolate–dextran (SPI–Dex)/sodium alginate (SA)-based external gelation water-in-oil-in-water (W/O/W) emulsions via a two-step emulsification process. The final SPD-enriched solution obtained after food-grade enrichment was used as the internal aqueous phase (W1). NaCl was added to W1 at a final concentration of 0.5% (*w*/*w*), and SPD was adjusted to four levels, namely 0.1%, 0.25%, 0.5%, and 1.0%, based on the mass of W1. The oil phase (O) consisted of soybean oil containing 6% (*w*/*w*, based on oil phase) PGPR. After magnetic stirring at room temperature for 20 min, a homogeneous oil phase was obtained. The external aqueous phase (W2) was prepared using soy protein isolate (SPI) and dextran (Dex) at an SPI:Dex mass ratio of 1:3 and a total solids content of 8% (*w*/*w*). NaCl was added to W2 at a final concentration of 0.5% (*w*/*w*), followed by the addition of sodium alginate at 0%, 0.25%, 0.5%, 0.75%, and 1.0% (*w*/*w*, based on W2 mass). After stirring at room temperature for 4 h, the resulting outer aqueous phase was stored at 4 °C overnight to allow sufficient hydration of the SPI–Dex matrix and uniform distribution of SA within the continuous phase. The selected SPD levels (0.1%, 0.25%, 0.5%, and 1.0%, *w*/*w* based on W1) were chosen to cover a representative low-to-high loading range, because the loading level of hydrophilic compounds in W/O/W emulsions can affect internal-phase retention and overall stability [13]. The selected SA levels (0, 0.25%, 0.5%, 0.75%, and 1.0%, *w*/*w* based on W2) included an SA-free control and a range sufficient to induce measurable changes in external-phase structuring and viscosity, since alginate concentration is closely related to the viscoelasticity and structural strength of polysaccharide-based emulsion systems [14]. In preliminary screening, higher SPD or SA levels were not further adopted because they led to less favorable formulation characteristics. Specifically, higher SPD levels were associated with a noticeable increase in amine-like odor, which is consistent with the odor-active nature of polyamines [15]; higher SA levels markedly reduced emulsion fluidity and made homogenization and handling more difficult. Therefore, the selected concentration ranges were considered appropriate for balancing formulation feasibility with systematic evaluation of composition-dependent effects.

The primary emulsion (W1/O) was first prepared by homogenizing W1 and O at a mass ratio of 5:5 using a T18 digital ULTRA–TURRAX homogenizer (IKA–Werke GmbH and Co. KG, Staufen, Germany) at 8000 r/min for 4 min. The obtained primary emulsion was then mixed with W2 at a W1/O:W2 mass ratio of 5:5 and further homogenized at 12,000 r/min for 5 min to obtain the external gelation W/O/W emulsion. After emulsification, the samples were allowed to stand at room temperature for 15 min to remove entrapped air bubbles. The total formulation weight of each treatment group was controlled at 20 g. The prepared emulsions were used for the determination of microstructure, particle size, zeta potential, rheological properties, encapsulation efficiency, retention efficiency, and storage stability, and part of each sample was further freeze-dried to obtain SPD-loaded microcapsules.

### 2.5. Preparation of Freeze-Dried Microcapsules

The freshly prepared external gelation W/O/W emulsions were allowed to stand for deaeration and then transferred into freeze-drying trays. After being evenly spread, the samples were pre–frozen under low-temperature conditions. Once completely frozen, they were lyophilized using an XSD–FD–20 freeze dryer (Jiangsu Bolaike Freezing Technology Development Co., Ltd., Changzhou, China). After lyophilization, the dried products were lightly milled, sealed in light–protected containers, and stored at 4 °C prior to analysis. The resulting freeze-dried powders were used for subsequent analyses of the physicochemical properties, encapsulation efficiency, loading capacity, microstructure, and storage stability of the microcapsules.

### 2.6. Characterization of Emulsions

#### 2.6.1. Microstructural Observation

To reduce droplet distortion caused by compression, a 10 μL sample of freshly made W/O/W emulsion was put onto a clean glass slide and carefully covered with a coverslip. The sample was left undisturbed for 5–10 min at room temperature to facilitate bubble dissipation and promote uniform spreading of the emulsion on the slide surface [16]. The emulsion microstructure was subsequently observed using a DMIL LED FLUO optical microscope (Leica Microsystems CMS GmbH, Wetzlar, Germany), and images were recorded under a 40× objective lens to evaluate droplet distribution and morphological characteristics. Micrographs were acquired and processed using the image acquisition and analysis software integrated with the instrument, and were used to characterize droplet dispersion uniformity, particle size distribution, and the possible occurrence of aggregation or coalescence within the emulsion system.

#### 2.6.2. Determination of Particle Size and Polydispersity Index (PDI)

A modified approach based on Liang et al. was used to assess particle size and PDI [17]. A Zetasizer Nano ZS particle size and zeta potential analyzer (Malvern Instruments Ltd., Malvern, UK) was used to measure the emulsion samples after they had been diluted 100 times with deionized water. The instrument was calibrated in advance using a blue–light calibrator (Malvern Instruments Ltd., Malvern, UK), and the refractive indices for distilled water and the W/O phase were assigned as 1.33 and 1.46, respectively. If the transmittance was higher than 75% after sample loading, the concentration was adjusted accordingly. Measurements were started when the obscuration remained within 8–12%, and the instrument response was stable. For every sample, measurements were made in triplicate, and the outcomes were presented as mean values.

#### 2.6.3. Determination of Zeta Potential

Fresh emulsions were diluted with distilled water to 0.02% solids and mixed thoroughly. A Zetasizer Nano ZS particle size and zeta potential analyzer (Malvern Instruments Ltd., Malvern, UK) was used to measure the zeta potential at 25 °C. Mean values were provided after each sample was examined three times.

#### 2.6.4. Determination of Rheological Properties

Rheological properties were determined according to Esteghlal et al. [18]. Fresh emulsion was placed at the center of the rheometer plate. The measuring geometry was then lowered to the trimming position, and excess sample was removed. A 40 mm stainless-steel cone-plate geometry with a cone angle of 4.0° was used. A Peltier plate was used to control the temperature. The truncation gap, loading gap, and trim gap offset were 500 μm, 550 μm, and 50 μm, respectively. All tests were performed at 25 °C. Before the test, the sample was allowed to stand on the plate for 60 s to reach thermal equilibrium. Steady shear tests were carried out using a logarithmic flow sweep. The shear rate ranged from 0.01 to 100 s^−1^, with 5 points per decade. At each point, the equilibration time was 5 s, and the averaging time was 30 s. Each sample was tested in triplicate.

The apparent viscosity (*η*) as a function of shear rate (*γ*) was recorded to evaluate the flow behavior of the emulsions. To quantitatively describe the shear-thinning behavior, the apparent viscosity–shear rate data were fitted using the Power Law model:

(5) $$\eta =K\gamma^{n-1}$$

where *η* is the apparent viscosity (Pa·s), *γ* is the shear rate (s^−1^), *K* is the consistency index (Pa·s^n^), and *n* is the flow behavior index. The goodness of fit was evaluated using the coefficient of determination (R^2^). The fitted parameters *K* and *n*, together with the apparent viscosity curves, were used to compare the effects of SPD loading and SA concentration on the rheological behavior of the emulsion systems.

#### 2.6.5. Determination of Encapsulation Efficiency (EE) and Retention Efficiency (RE)

Two emulsion aliquots (*m*_0_ g each) were used for EE and RE determination following a modified Batista et al. method [19]. After centrifugation at 3000 r/min (960× *g*) for 5 min, the supernatant from one aliquot was collected for free SPD measurement. The supernatant was collected for the total SPD measurement after the other aliquot was ultrasonically disturbed (80% amplitude, 130 W, 3 s on/2 s off, 100 s total) and centrifuged for 10 min at 12,000 r/min (15,292× *g*). A 0.22 μm aqueous membrane was used to filter all supernatants prior to HPLC analysis. The concentrations of free and total SPD were represented by the symbols *c*_1_ and *c*_2_ (g/mL), respectively. The following formulas were used to determine EE and RE:

(6) $$EE\left(\%\right)=1-\frac{c_{1}}{c_{2}}\times 100$$

(7) $$RE\left(\%\right)=\frac{c_{2}\times V}{m_{0}\times x_{0}}\times 100$$

where *c*_1_ is the mass concentration of free SPD in the emulsion (g/mL), *c*_2_ is the mass concentration of total SPD in the emulsion (g/mL), *V* is the total volume of the extracted solution (mL), *m*_0_ is the total mass of the emulsion sample (g), and *x*_0_ is the theoretical addition level of SPD (g/g).

#### 2.6.6. Determination of Emulsion Storage Stability

Freshly prepared emulsion samples were transferred into transparent glass tubes and stored at 25 °C for 7 days under static conditions. During storage, the samples were photographed to visually monitor creaming, layering, and phase separation. To quantitatively evaluate the storage stability of the emulsions, the creaming index (*CI*) was calculated according to the height of the separated serum layer after storage. The total height of the emulsion column and the height of the lower clear serum layer were recorded as *H_t_* and *H_s_*, respectively. The layer heights were measured directly from the sample tubes using a ruler or from digital images using image analysis software. The *CI* was calculated using the following equation:

(8) $$CI\left(\%\right)=\frac{H_{s}}{H_{t}}\times 100$$

where *H_s_* is the height of the separated serum layer, and *H_t_* is the total height of the emulsion sample. A lower *CI* value indicates better resistance to phase separation and higher storage stability. All measurements were performed in triplicate.

### 2.7. Characterization of Microcapsules

#### 2.7.1. Fourier Transform Infrared Spectroscopy Analysis (FTIR)

FTIR spectra of SPD, PGPR, SPI, Dex, SA, NaCl, and freeze-dried microcapsules were recorded on an iS50 FTIR spectrometer equipped with an attenuated total reflectance accessory (Thermo Fisher Scientific Inc., Waltham, MA, USA). Spectra were collected over 400–4000 cm^−1^ at 4 cm^−1^ resolution with 32 scans per sample [16]. Structural features of the microcapsules and interactions among the components were evaluated from changes in the position and intensity of characteristic absorption bands.

#### 2.7.2. Scanning Electron Microscopy Observation

An SU8100 cold field-emission scanning electron microscope (Hitachi, Tokyo, Japan) was used to examine the microstructure of the microcapsules [20]. Samples were mounted on specimen stubs, sputter-coated with gold, and imaged at 200× magnification.

#### 2.7.3. Determination of Moisture Content and Water Activity

Moisture content was determined by oven drying [21]. Samples were weighed into pre-dried aluminum dishes and dried at 105 °C. After 2 h, the dishes were cooled in a desiccator for 30 min and weighed, followed by repeated drying at 105 °C for 1 h intervals until constant weight was reached. Moisture content was calculated as follows:

(9) $$\mathrm{Moisture}\;\mathrm{content}\;(\%)=\frac{m_{1}-m_{2}}{m_{1}-m_{0}}\times 100$$

where *m*_0_ is the mass of the aluminum dish (g), *m*_1_ is the total mass of the dish and sample before drying (g), and *m*_2_ is the total mass of the dish and sample after drying to constant weight (g).

The water activity of the microcapsule powders was determined with a LabSwift-aw water activity meter (Novasina AG, Lachen, Switzerland). Prior to measurement, the instrument was calibrated using manufacturer-supplied reusable SAL–T humidity standards (11%, 58%, and 84% relative humidity (RH); Novasina AG, Lachen, Switzerland). They were then placed in the designated sample cup and measured at room temperature. The result was recorded once the reading stabilized and the instrument indicated completion. Water activity and moisture content were determined from the same batch of freeze-dried microcapsules. All formulations were prepared from three independent freeze-drying runs, and each measurement was performed in triplicate. Results are reported as mean ± SD.

#### 2.7.4. Determination of Encapsulation Efficiency and Retention Efficiency

The free and total SPD contents in the freeze-dried microcapsules were determined by HPLC, and accordingly, the encapsulation and retention efficiencies were computed [19]. Two portions of microcapsule samples, each weighing *m*_0_ g, were accurately transferred into centrifuge tubes and dispersed thoroughly in 5 mL of ultrapure water (18.2 MΩ·cm at 25 °C). One portion was centrifuged at 3000 r/min (956× *g*) for 5 min, and the supernatant was collected for the determination of free SPD. The other portion was subjected to ultrasonic disruption under the following conditions: amplitude, 80%; power, 130 W; pulse-on time, 3 s; pulse-off time, 2 s; and total sonication time, 100 s. The disrupted sample was then centrifuged at 12,000 r/min (15,292× *g*) for 10 min, and the supernatant was collected for the determination of total SPD. All supernatants were membrane-filtered (0.22 μm) and analyzed under the HPLC conditions described in Section 2.3. The concentrations of free SPD and total SPD were recorded as *c*_1_ and *c*_2_ (g/mL), respectively.

EE and RE of the microcapsules were calculated as follows:

(10) $$EE\left(\%\right)=1-\frac{c_{1}}{c_{2}}\times 100$$

(11) $$RE\left(\%\right)=\frac{c_{2}\times 5}{m_{0}\times x_{0}}\times 100$$

where *c*_1_ is the mass concentration of free SPD in the microcapsules (g/mL), *c*_2_ is the mass concentration of total SPD in the microcapsules (g/mL), *m*_0_ is the mass of the microcapsule sample (g), and *x*_0_ is the theoretical addition level of SPD (g/g).

### 2.8. Data Processing and Statistical Analysis

All experiments were performed in at least three independent replicates, and results are reported as mean ± standard deviation (SD). Normality and homogeneity of variance were assessed using the Shapiro–Wilk and Levene’s tests. Differences among groups were analyzed by one-way analysis of variance (ANOVA) followed by Tukey’s honestly significant difference (HSD) test, with *p* < 0.05 considered significant. Statistical analyses and figures were generated using Origin 2025 (OriginLab Corp., Northampton, MA, USA) and SPSS 27.0 (IBM Corp., Armonk, NY, USA).

## 3. Results and Discussion

### 3.1. Recovery Efficiency and Enrichment Performance of SPD from Beech Mushroom Cooking Liquor

The quantitative HPLC results are presented in Table S1 in the Supplementary Materials. The reliability of SPD quantification was supported by the HPLC method validation described in Section 2.3. The calibration showed good linearity over 1–1000 mg/L, with R^2^ = 0.9937. The LOD and LOQ were 0.13 mg/L and 0.40 mg/L, respectively. The concentration of SPD in the beech mushroom cooking liquor was 69.17 mg/L. Based on an initial cooking liquor volume of 500 mL, the total amount of SPD was estimated to be approximately 34.59 mg. This finding indicates that beech mushroom processing by-products may serve as a practical source of SPD. This result is consistent with previous reports showing that mushrooms are relevant dietary sources of spermidine. For example, Jabłońska-Ryś et al. [22] reported spermidine contents of 266.47 ± 13.38 and 367.22 ± 14.19 mg/kg in unprocessed white and brown button mushrooms, respectively. Buyukuslu et al. [23] also reported mushrooms as spermidine-rich foods, with a spermidine concentration of 88.6 mg/kg. Because these values refer to mushroom tissues rather than cooking liquor, direct numerical comparison should be interpreted with caution. Nevertheless, these reports support the feasibility of recovering SPD from mushroom-derived matrices.

After filtration, the recovery of SPD remained as high as 96.38%, suggesting that it was predominantly present in dissolved form in the liquid phase and that the filtration step caused only minor losses. Following vacuum concentration at below 40 °C to one-tenth of the original volume, the SPD concentration increased to 525.60 mg/L, whereas the overall recovery decreased to 75.98%. This result indicates that although the concentration process substantially enhanced the level of the target compound, a certain degree of operational loss was unavoidable. The loss of SPD may be related to repeated transfer, concentration, redissolution, and filtration. Partial adsorption of amine-containing molecules on membranes, glassware, centrifuge tubes, or other contact surfaces may also have occurred. In addition, part of the SPD may have been retained in precipitated impurities during ethanol treatment and centrifugation. Such losses are consistent with the transfer loss commonly observed for water-soluble low-molecular-weight compounds after their migration into the liquid phase during thermal processing, and may also be associated with the adsorption of amine-containing molecules onto container surfaces or analytical and processing interfaces [24]. After further clarification by centrifugation, the SPD concentration in the supernatant was 520.10 mg/L, with a final overall recovery of 72.16%. The additional reduction in total amount during centrifugation suggests that part of the SPD may have been removed together with the precipitate or lost during supernatant collection. Nevertheless, this treatment improved the clarity and homogeneity of the enriched solution, thereby providing a more stable internal aqueous phase for subsequent emulsion construction [24]. The final enrichment factor was 7.52, indicating that low-temperature concentration was the main contributor to SPD enrichment. The slight decrease from the concentration stage to the final enriched solution indicates that the subsequent clarification steps caused limited additional loss. SPD in beech mushroom cooking liquor could be effectively recovered through filtration, low-temperature concentration, and centrifugal clarification, further indicating the potential for value-added utilization of nitrogen-containing compounds present in thermal processing by-products of beech mushroom.

### 3.2. Microstructural Characteristics of External Gelation W/O/W Emulsions

**Figure 1:**
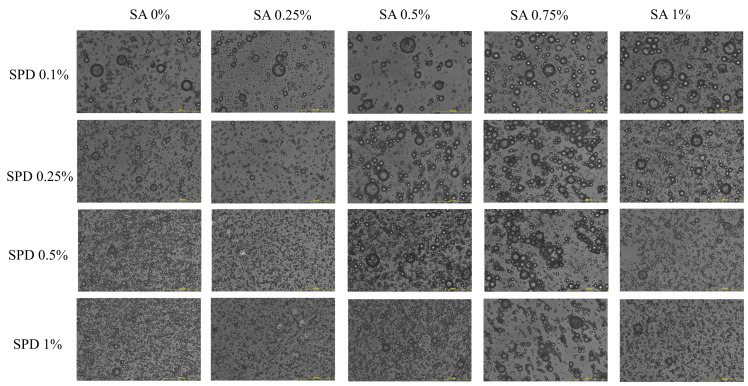
Microstructure of external gelation W/O/W emulsions with different SPD and SA combinations.

For W/O/W double emulsion systems, successful formation can be confirmed under optical microscopy when a typical multiple structure is observed, in which several small droplets are encapsulated within larger outer droplets, with clear droplet boundaries, uniform distribution, and no obvious aggregation or coalescence [25]. As shown in Figure 1, all prepared external gelation W/O/W emulsions exhibited a characteristic multiple-droplet dispersed structure, which is consistent with the hierarchical organization of the inner aqueous phase, oil layer, and outer aqueous phase generated through stepwise emulsification in double emulsions [26]. As the concentration of SA rose from 0% to 1%, the fraction of successfully encapsulated droplets increased across all SPD loading groups, suggesting enhanced microstructural organization of the emulsion. Incorporating SA into the external aqueous phase also led to a more uniform droplet distribution and greater physical stability [16]. Across different SPD loading levels, as SPD increased from 0.1% to 1.0%, the overall number of droplets observed in the microscopic field increased, suggesting that increasing internal aqueous phase loading promoted the formation of multiple-droplet structures. However, at each SPD level, the overall trend of progressive droplet refinement with increasing SA remained unchanged, indicating that structural regulation by the outer aqueous phase was the dominant factor in this system [27]. Mechanistically, SA mainly affected the secondary emulsification process, raising the viscosity of the continuous phase, modifying the viscosity balance between phases, and limiting droplet migration and recoalescence [28]. Protein–polysaccharide composite systems can simultaneously form a thicker, more stable interfacial layer around droplets, increasing emulsion resistance to flocculation and coalescence via steric and electrostatic effects. Therefore, the coexistence of SPI–Dex and SA facilitated the formation of droplet structures with clear boundaries and pronounced multiple-emulsion characteristics [8].

### 3.3. Analysis of Particle Size, PDI, and Zeta Potential of the Emulsions

The particle size, PDI, and ζ-potential of the W/O/W emulsions are shown in Table S2. Particle size was affected by both SPD loading and SA concentration, but it did not show a simple monotonic relationship with SA concentration. In the SPD 0.1% and 0.25% groups, particle size generally increased with increasing SA level. For example, in the SPD 0.25% group, particle size increased from 2775.00 nm at 0% SA to 9013.67 nm at 1.0% SA. However, this trend was not observed in all formulations. In the SPD 0.5% group, particle size increased to 10,273.33 nm at 0.75% SA but decreased to 3498.33 nm at 1.0% SA. In the SPD 1.0% group, particle size remained relatively low within 0–0.75% SA and reached the minimum value of 2482.67 nm at 0.5% SA. These results indicate that droplet size was regulated by both internal-phase loading and external-phase composition. This is consistent with the structural complexity of W/O/W double emulsions, in which droplet size is affected by inner droplet encapsulation, secondary emulsification, and interfacial composition [29].

PDI was included to further evaluate the width of the particle size distribution. The PDI values ranged from 0.36 to 0.93, indicating that several formulations had broad droplet size distributions. In the low-SPD groups, PDI values were generally high. The SPD 0.1% group showed PDI values of 0.66–0.93, and the SPD 0.25% group showed values of 0.71–0.91. By contrast, lower PDI values were observed in the SPD 1.0% group at 0.5% and 0.75% SA, with values of 0.36 and 0.43, respectively. The SPD 0.5% SA 1.0% group also showed a relatively low PDI value of 0.41. Since PDI reflects the breadth of particle size distribution, these results indicate that mean particle size alone was not sufficient to evaluate emulsion quality [30]. Combined with the optical micrographs in Figure 1, formulations with larger particle sizes and higher PDI values tended to show more heterogeneous droplet populations, whereas formulations with lower PDI values showed relatively more uniform droplet dispersion.

The ζ-potential results further described the surface charge characteristics of the emulsions. Without SA, all groups showed relatively low absolute ζ-potential values, ranging from −12.13 to −20.77 mV. After the SA addition, the absolute ζ-potential increased in most groups. For instance, the SPD 0.25% group reached −36.67 mV, and the SPD 0.1% group reached −35.20 mV at 0.75% SA. This change can be attributed to the anionic character of SA and the contribution of carboxyl groups to the droplet surface or continuous phase [31,32]. However, the ζ-potential also showed formulation-dependent variation. In the SPD 0.1% and 0.25% groups, the absolute ζ-potential decreased at 1.0% SA compared with 0.75% SA. This result indicates that surface charge was not determined only by SA concentration. Interfacial rearrangement or charge shielding may have contributed to this non-monotonic change.

The combined analysis of particle size, PDI, ζ-potential, and optical microstructure indicates that emulsion properties depended on the balance between SPD loading and SA concentration. An appropriate SPD–SA combination improved droplet distribution and surface charge characteristics, whereas excessive or insufficient SA did not necessarily produce smaller or more uniform droplets.

### 3.4. Rheological Properties of the Emulsions

**Figure 2:**
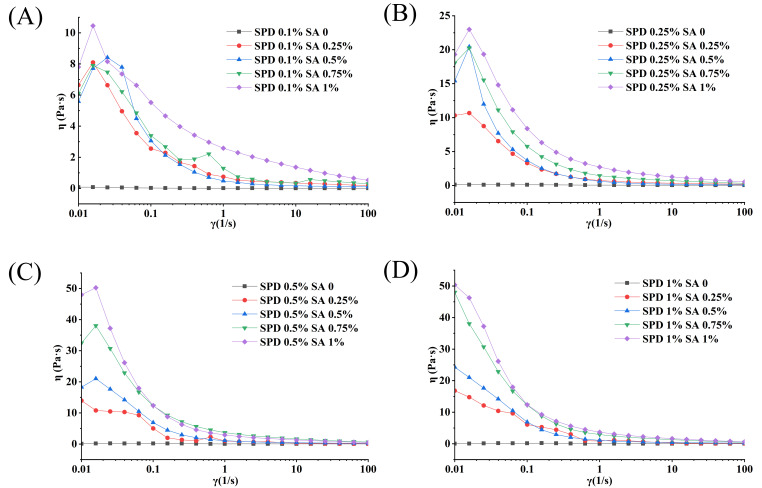
Apparent viscosity–shear rate curves of external gelation W/O/W emulsions with different SPD and SA combinations. (**A**) SPD 0.1%; (**B**) SPD 0.25%; (**C**) SPD 0.5%; (**D**) SPD 1.0%.

As shown in Figure 2, all SPI–Dex/SA external gelation W/O/W emulsions exhibited shear-thinning behavior, as the apparent viscosity decreased with increasing shear rate. To quantify this behavior, the apparent viscosity–shear rate data were fitted using the Power-law model, and the fitted parameters are shown in Table S3. All formulations showed n < 1, confirming their non-Newtonian pseudoplastic behavior. The R^2^ values ranged from 0.9733 to 0.9966, indicating that the Power-law model adequately described the steady shear behavior of the emulsions. Similar use of the Power-law model has been reported for food emulsions and emulsion-like systems to quantify shear-thinning behavior and compare flow properties [33]. The consistency index K increased with increasing SA concentration within each SPD level. In the SPD 0.1% group, K increased from 0.0320 at 0% SA to 11.6201 at 1.0% SA. Similar increases were observed in the SPD 0.25%, 0.5%, and 1.0% groups, where K increased from 0.0738 to 10.0030, from 0.0861 to 4.3661, and from 0.0216 to 15.4875, respectively. These results indicate that SA increased the resistance of the emulsions to flow. This trend is consistent with previous reports showing that sodium alginate can increase the viscosity of the continuous phase and improve the rheological stability of the emulsion system [34]. The flow behavior index n showed formulation-dependent variation. In the SPD 0.1% and 0.25% groups, n generally increased with SA concentration, whereas in the SPD 0.5% and 1.0% groups, n changed non-monotonically. This indicates that shear-thinning behavior was affected by both SPD loading and SA concentration, rather than by SA alone. Similar formulation-dependent rheological responses have also been reported in protein–polysaccharide emulsion systems, where polysaccharide concentration, interfacial composition, and homogenization conditions jointly affect flow behavior [35].

The effect of SPD became more evident when SA was present at medium or high levels. In particular, the SPD 1.0% series showed relatively high K values at 0.5–1.0% SA, suggesting greater emulsion consistency under these formulation conditions. This may be associated with increased internal aqueous phase loading and possible ionic association between SPD and alginate. Alginate–spermidine systems based on ionic interaction have been reported previously [36]. However, because oscillatory rheological measurements and direct interaction assays were not performed in the present study, this explanation should be considered a possible mechanism rather than direct experimental evidence. Accordingly, the rheological results are interpreted here as evidence of changes in steady-flow behavior and consistency, rather than as direct proof of a three-dimensional viscoelastic network. The combined analysis of Figure 2 and Table S3 shows that SA increased the flow resistance of the W/O/W emulsions, while SPD loading modulated the rheological response depending on SA level. These results provide quantitative support for formulation-dependent changes in emulsion flow behavior.

### 3.5. Analysis of Encapsulation Efficiency and Retention Efficiency of the Emulsions

As shown in Table 1, the SPI–Dex/SA external gelation W/O/W emulsion system exhibited a strong capacity for SPD entrapment, with encapsulation efficiency values above 92.90% for all treatment groups. This result indicates that the system effectively restrained leakage of the hydrophilic small-molecule SPD into the outer phase during secondary emulsification. In W/O/W double emulsions, water-soluble bioactives are retained in the inner aqueous phase, whereas the oil layer, together with the outer aqueous phase, forms multiple barriers that improve encapsulation efficiency and delivery stability [37]. Further comparison showed that the improvement in encapsulation efficiency was primarily driven by SPD concentration, whereas the role of SA was mainly auxiliary and stabilizing. When the SPD level increased from 0.1% to 1.0%, encapsulation efficiency rose significantly from 92.90 to 95.12% to 99.68–99.76%, indicating that, within the formulation range investigated in this study, the loading capacity of the wall material had not yet reached saturation. Instead, higher core loading reduced the relative proportion of fixed leakage relative to the total amount added, thereby increasing the apparent encapsulation efficiency. Meanwhile, at the same SPD level, the addition of SA generally contributed to maintaining a high encapsulation efficiency, and this effect was more pronounced under low-SPD conditions. For example, in the SPD 0.1% group, encapsulation efficiency rose from 92.95% to 95.12% as the SA concentration increased from 0 to 1%, indicating that structuring and gelation of the external aqueous phase reduced solute migration between the internal and external phases. This finding aligns with previous reports showing that external-phase gelation enhances the encapsulation stability of double emulsions [38]. In addition, as a cationic polyamine, SPD may be further constrained within the network through ionic interactions with alginate, which is in agreement with the interaction behavior previously observed in alginate–spermidine composite gel systems [36]. Compared with encapsulation efficiency, the retention efficiency of all treatment groups remained within the range of 92.47–96.87%, with relatively small variations among groups, indicating that different SPD and SA gradients exerted only limited effects on the final preservation of SPD during subsequent processing. Taken together, the SPI–Dex/SA external gelation W/O/W emulsion system not only achieved efficient encapsulation and high retention of beech mushroom-derived SPD, but also demonstrated that the structural barrier formed by the synergistic combination of a protein–polysaccharide composite interface and external-phase gelation was beneficial for reducing the direct exposure of SPD, thereby providing experimental support for converting bitterness-related compounds in beech mushroom thermal processing by-products into functional delivery carriers [37].

### 3.6. Analysis of Storage Stability of the Emulsions

**Figure 3:**
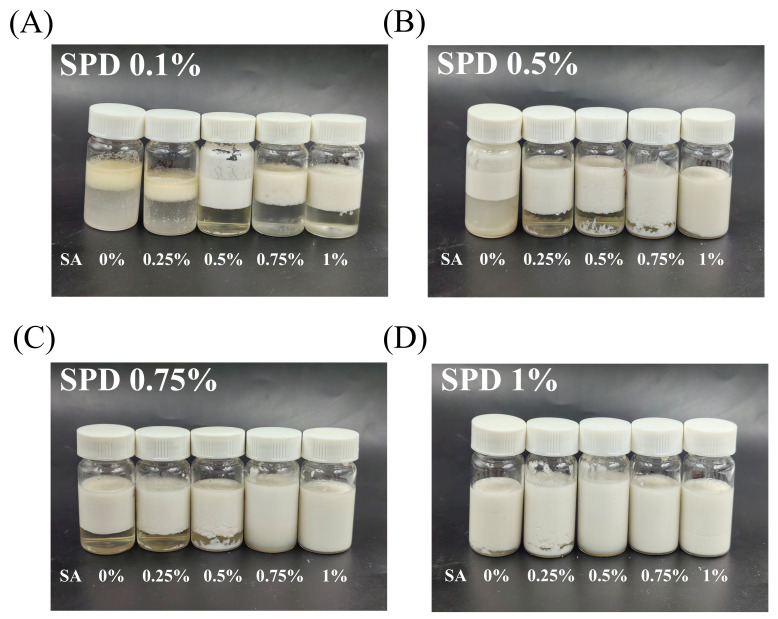
Visual appearance of external gelation W/O/W emulsions with different SPD and SA combinations after 7 days of storage at room temperature. (**A**) SPD 0.1%; (**B**) SPD 0.25%; (**C**) SPD 0.5%; (**D**) SPD 1.0%.

As shown in Figure 3, after 7 days of storage at room temperature, all treatment groups exhibited different degrees of upper creaming, lower serum separation, or bottom sedimentation. These changes indicated that the W/O/W emulsions underwent physical destabilization during storage, which is commonly associated with gravity-driven phase separation and droplet migration in emulsion systems [29]. The creaming index (CI) was further calculated to quantify phase separation, and the results are shown in Figure S1. A lower CI value represents better storage stability. The CI values showed marked differences among formulations. The SPD 0.1% group showed the highest CI values, ranging from approximately 32% to 63%. This result indicates that low SPD loading was not favorable for maintaining emulsion stability. Although increasing SA concentration reduced CI in this group, the CI remained relatively high even at 1.0% SA. This trend was consistent with the visual appearance in Figure 3, where the low-SPD samples showed more pronounced phase separation [39]. An increasing SPD loading markedly reduced CI. In the SPD 0.25% group, CI decreased from approximately 47% at 0% SA to about 2% at 1.0% SA. In the SPD 0.5% group, CI remained at approximately 23–24% when SA was 0–0.5%, but decreased to about 6% and 2% at 0.75% and 1.0% SA, respectively. In the SPD 1.0% group, all formulations showed low CI values, and the CI was close to or below 1% when SA was added at 0.5–1.0%. These results indicate that SPD loading had an important effect on storage stability, while sufficient SA addition further reduced serum separation. The improvement in storage stability may be associated with changes in droplet distribution, external aqueous phase composition, and interfacial charge. As discussed in Section 3.2 and Section 3.3, SA affected both the microstructure and ζ-potential of the emulsions. In most formulations, SA increased the absolute ζ-potential, which may have enhanced electrostatic repulsion between droplets and reduced droplet aggregation [40]. However, the CI results indicate that SA concentration alone did not determine storage stability. For example, in the SPD 0.5% group, CI changed only slightly from 0% to 0.5% SA, but decreased sharply at 0.75% and 1.0% SA. Therefore, the stabilizing effect of SA depended on the SPD level and the overall formulation composition. The CI data were also partly consistent with the particle size and PDI results. Formulations with high CI values generally showed broader particle size distributions or less uniform droplet dispersion, whereas formulations with low CI values tended to show better resistance to phase separation. Larger droplets or droplet aggregates are more susceptible to gravitational separation, which can promote creaming or sedimentation [41]. Nevertheless, the storage behavior should be interpreted using the combined results of visual appearance, CI, particle size, PDI, and ζ-potential, rather than from a single parameter. Although protein–polysaccharide systems can improve interfacial stability, droplet aggregation or flocculation may still reduce long-term stability when the formulation is not properly balanced [42].

To conclude, the combined analysis of Figure 3 and Figure S1 demonstrated that the storage stability of the SPD-loaded SPI–Dex/SA external gelation W/O/W emulsions was dependent on formulation composition. Higher SPD loading and appropriate SA addition reduced serum separation and improved storage stability, whereas low SPD loading resulted in relatively poor stability even after SA incorporation.

### 3.7. Surface Morphology of Freeze-Dried Microcapsules

**Figure 4:**
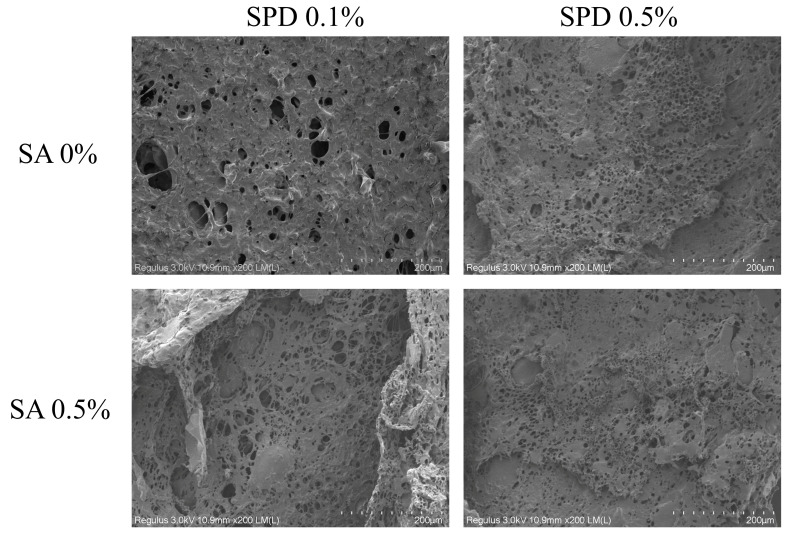
Scanning electron micrographs of freeze-dried microcapsules prepared with different SPD and SA combinations.

Scanning electron microscopy (SEM) provides qualitative information on surface structural changes in food matrices through analysis of the signals generated when the sample surface is irradiated and scanned by an electron beam [43]. As shown in Figure 4, different combinations of SPD and SA altered the microstructural morphology of the freeze-dried microcapsules. All samples exhibited a porous matrix structure, although differences were observed in pore size, overall pore morphology, and network continuity [44]. Under low-SPD conditions, the SPD 0.1%/SA 0% group exhibited larger pores and less compact pore morphology than the other groups. When the SA concentration increased to 0.5%, the sample developed a more distinct interconnected porous structure, indicating that the addition of SA promoted continuous-phase structuring and gel skeleton formation, thereby enhancing network support capacity and structural integrity [45]. At the same SA level, increasing SPD from 0.1% to 0.5% transformed the sample from a structure with relatively large and unevenly distributed pores into a denser porous network with smaller and more uniformly distributed pores, suggesting that higher SPD loading was conducive to improving matrix filling and local structural integrity. Jia et al. [46] similarly reported that protein–polysaccharide composite wall materials can form more stable microstructures under appropriate loading conditions. However, although the SPD 0.1%/SA 0.5% group exhibited more pronounced network characteristics than the low-SA groups, large cavities and local fractures were still present, indicating that simply increasing SA primarily enhanced gel formation and skeleton establishment, while its ability to improve structural uniformity under low-loading conditions remained limited. Studies on double emulsions have likewise shown that the structural stabilization effect of gelling agents depends on their compatibility with internal-phase loading and interfacial conditions [26]. By contrast, the SPD 0.5%/SA 0.5% group formed the finest and most continuous porous structure, with a marked reduction in large cavities. This result is consistent with its relatively high encapsulation efficiency and superior storage stability, indicating that the combination of a moderate SA network and higher SPD loading promoted more effective structural confinement [47]. Increasing SA also enhanced the surface negative charge of the system, while ionic interactions between alginate and SPD may jointly influence network organization and core retention capacity [36]. Therefore, the synergistic effect of SPD and SA was mainly reflected in strengthening the continuous-phase structure, improving local confinement, and reducing core migration, all of which favored the formation of a more stable encapsulation matrix [48].

### 3.8. Analysis of Particle Size, PDI, and Zeta Potential of the Microcapsules

Table S4 shows the particle size, PDI, and ζ-potential of freeze-dried microcapsules prepared with different SPD and SA ratios. In the SPD 0.1% group, particle size increased from 5080.00 nm to 7591.67 nm as the SA level rose from 0% to 1%, indicating that under low-loading conditions, higher SA mainly promoted thickening of the outer wall matrix and expansion of the gel network. In general, increasing SA concentration is often accompanied by simultaneous increases in particle size and swelling capacity [36]. Under high-SPD conditions, the particle size of the SPD 1% group remained within the range of 6452.67–7227.00 nm when the SA concentration was between 0% and 0.75%, and increased to 8077.00 nm only at 1% SA. This result indicates that higher core loading improved the consistency of particle formation to some extent, whereas a moderate wall material level was more favorable for obtaining freeze-dried particles with relatively stable dimensions. This trend is in agreement with the previously observed encapsulation efficiency and FTIR results, since SPD can interact ionically with SA to form composite micro-/nanogels, thereby enhancing local network confinement and reducing disordered aggregation during particle formation [49]. At the same time, the increase in particle size observed in some groups under high-SA conditions suggests that when the polysaccharide level was excessive, the system may have exhibited a larger apparent particle size because of overdevelopment of the network, bridging effects, or post-freeze-drying aggregation. In addition, the freeze-drying process itself may preserve or even amplify pre-existing aggregate structures [50]. The PDI values of the re-dispersed freeze-dried microcapsules ranged from approximately 0.20 to 0.45, indicating a moderate particle size distribution after reconstitution. The variation in PDI did not follow the trend observed for particle size, suggesting that distribution uniformity was not solely determined by particle size, but was also influenced by aggregation during freeze-drying and re-dispersion behavior. In the SPD 0.1% group, PDI values slightly decreased with increasing SA concentration, indicating improved distribution uniformity at higher SA levels. In contrast, relatively higher PDI values were observed at low SA levels (0–0.25%) in the SPD 0.25% and 0.5% groups, suggesting insufficient stabilization and a higher tendency for aggregation. As the SA concentration increased, the PDI values decreased, indicating improved dispersion behavior. In the SPD 1% group, PDI values remained at a moderate level overall, with slightly higher values at low SA levels and reduced values at 0.75% SA. Overall, a moderate SA level combined with medium-to-high SPD loading was more conducive to obtaining microcapsules with relatively appropriate particle size and stable structure.

From the changes in the data, it can be seen that in the SPD 0.1% group, as the SA concentration increased from 0% to 1%, the zeta potential gradually decreased from −26.03 mV to −44.30 mV, indicating that SA was the primary factor determining the surface charge characteristics of the microcapsules. In addition, the formation of the protein–polysaccharide composite wall further influenced surface charge distribution and particle stability [51]. Together with the particle size results, these findings indicate that increasing SA favored enhancement of the surface negative charge density of the microcapsules, whereas increasing SPD concentration regulated this process through its interaction with SA. This is in line with previous findings in microsphere-based delivery systems, in which microsphere composition and structural characteristics were shown to affect moisture-related behavior, swelling, and release performance [52].

### 3.9. Infrared Spectral Analysis of Raw Materials and Microcapsules

**Figure 5:**
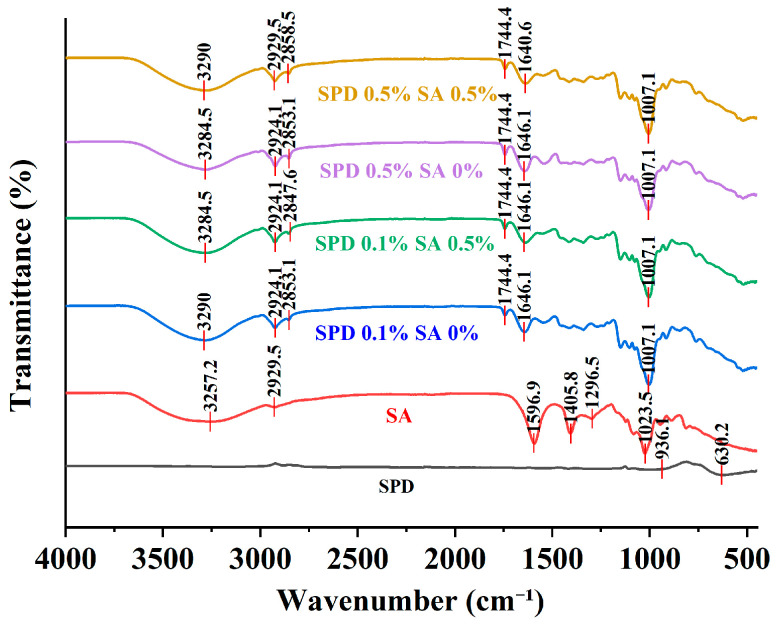
FTIR spectra of raw materials and freeze-dried microcapsules.

As shown in Figure 5, SA displayed a broad absorption band near 3257.2 cm^−1^, characteristic peaks at 1580.9 and 1402.2 cm^−1^, and a distinct band around 1032.5 cm^−1^. These peaks are attributed to O–H stretching, the asymmetric and symmetric stretching vibrations of carboxylate COO^−^ groups, and the C–O–C vibration of glycosidic linkages in polysaccharides, respectively [53]. All composite microcapsule samples displayed broad absorption bands in the range of 3284.5–3290 cm^−1^. Compared with SA alone, these peaks showed both positional shifts and band broadening, indicating that the hydrogen-bonding environment surrounding hydroxyl and amino groups was reorganized after complex formation. In protein–polysaccharide systems, such spectral changes in the high-wavenumber region are generally regarded as evidence of enhanced hydrogen bonding and intensified intermolecular association [54]. In addition, the composite samples retained C–H stretching vibration bands near 2924.1 and 2853.1–2847.6 cm^−1^, indicating that the freeze-drying and encapsulation processes did not destroy the principal organic backbone of the system, and that the observed structural changes mainly originated from intermolecular interactions rather than degradation of the polymer main chains [55].

Changes in the mid-wavenumber region further confirmed the existence of pronounced interactions between the wall materials and the core substance. The composite samples showed a stable absorption band near 1646.1 cm^−1^, a region generally associated with overlapping contributions from the protein amide I band, bending vibration of bound water, and certain carbonyl-related vibrations. Accordingly, changes in peak shape and position in this region usually reflect alterations in the local protein conformation and the microenvironment of carbonyl groups [54]. Considering the composition of the present system, it may be inferred that SPI–Dex, SA, and SPD formed a composite network dominated by hydrogen bonding and electrostatic interactions, thereby altering the molecular arrangement around amide and carboxyl groups [56]. SA and spermidine can form micro- or nanogels through ionic interactions, and FTIR can capture the corresponding spectral changes induced by such interactions [36]. In the low-wavenumber region, all composite samples exhibited a strong absorption peak near 1007.1 cm^−1^, indicating that the polysaccharide backbone remained the principal structural support of the microcapsule network. Accordingly, SA and Dex played dominant roles in particle formation and network maintenance [55].

It is noteworthy that no obvious new strong characteristic peaks appeared in the composite samples. Instead, the spectra were mainly characterized by shifts, broadening, and intensity changes in existing absorption bands, indicating that no substantial formation of new covalent bonds occurred during microcapsule formation, and that SPD immobilization mainly relied on non-covalent interactions and confinement within the wall material network. In SA-based microcapsules and protein–polysaccharide encapsulation systems, such FTIR features generally indicate that the encapsulation mechanism is dominated by physical entrapment, hydrogen-bond association, electrostatic adsorption, and chain entanglement rather than chemical grafting or covalent crosslinking [57]. The incorporation of SA increased the viscosity of the continuous phase and improved the integrity of the polysaccharide network, SPI–Dex provided interfacial support, and SPD reduced its tendency toward diffusion and leakage through interactions with SA and the polar groups of the protein matrix, thereby jointly enhancing the encapsulation stability of the system [58]. Overall, the FTIR results confirmed that SPD was successfully embedded within the SPI–Dex/SA composite wall material network, and that the stabilization mechanism of the system was primarily derived from hydrogen bonding, electrostatic interactions, and the dense network structure cooperatively formed by polysaccharides and proteins.

### 3.10. Analysis of Moisture Content and Water Activity of the Microcapsules

As shown in Table S5, the water activity (aw) of the freeze-dried microcapsules in all treatment groups remained at a relatively low level (0.2139–0.2279), and the moisture content was also low overall (2.14–2.88%), indicating that the obtained powders exhibited good dehydration efficiency and favorable storage stability [59]. In general, the variation in aw was smaller than that in moisture content, suggesting that differences among formulations primarily affected the state of residual water rather than substantially altering the total moisture level of the samples. With changes in the proportions of SA and SPD, both aw and moisture content showed certain fluctuations, but no linear pattern was observed, indicating that these two parameters were jointly regulated by the wall material network structure, water-binding state, and pore characteristics formed after freeze-drying. Some samples maintained low aw despite having slightly higher moisture contents, suggesting that the residual water in these samples mainly existed as bound water rather than as mobile free water [60]. Based on the system composition, it may be inferred that polar groups such as proteins, polysaccharides, and carboxyl groups in the SPI–Dex/SA composite wall matrix enhanced water immobilization through hydrogen bonding and ionic interactions, thereby reducing the proportion of free water [61]. In addition, the regulatory effect of SA on network compactness and water-retention behavior may explain why aw and moisture content did not change synchronously among different formulations.

### 3.11. Analysis of Encapsulation Efficiency and Retention Efficiency of the Microcapsules

As shown in Table 2, the EE of the different treatment groups ranged from 56.44% to 98.13%, while the RE ranged from 70.21% to 89.12%. These results indicate that the composite wall material constructed from SPI–Dex and SA was able to further immobilize the internal-phase solute on the basis of the emulsion template and to form a relatively stable encapsulation structure after drying [49]. Polysaccharide hydrogels and protein–polysaccharide composite matrices can enhance the EE of bioactive compounds by improving network integrity, reducing diffusion rate, and increasing interfacial stability [62]. When the SPD level increased from 0.1% to 1.0%, the EE in the SPD 0.1% group was only 56.44–74.71%, whereas that in the SPD 0.5% and 1.0% groups increased to 86.40–96.54% and 94.67–98.13%, respectively. This finding suggests that, within the formulation range investigated in this study, the wall material system had not yet reached loading saturation. As the core concentration increased, the relative proportion of unavoidable surface loss or migration loss during preparation and analysis decreased with respect to the total amount added, resulting in a continuous increase in apparent EE. Gholivand et al. [63] reported that plant protein-based composite wall materials can exhibit high loading adaptability and significantly improve the EE of active compounds when the formulation ratio is appropriate. Regarding SA concentration, in the SPD 0.1% group, EE first increased from 56.44% to 74.71% and then declined to 66.95% with increasing SA concentration. In the SPD 0.25% group, the EE reached 87.03% at 0.5% SA, which was clearly higher than that of the other treatments. In the SPD 0.5% group, EE increased with SA concentration to 96.54% and then decreased slightly, whereas in the SPD 1.0% group, the highest value of 98.13% was obtained at 0.25% SA. These results indicate that SA mainly functioned by regulating wall-matrix compactness, improving particle structural integrity, and strengthening core confinement, rather than merely increasing loading capacity [51]. SA-containing microcapsule systems generally improve structural stability and retard the release of active compounds, although such effects usually occur within an optimal addition range, and excessive addition does not necessarily lead to further improvement. On the other hand, the RE of all treatment groups fluctuated within the range of 70.21–89.12%, with no significant differences observed. This indicates that formulation changes in the present study mainly affected the initial degree of encapsulation, while exerting relatively limited influence on protection during the subsequent drying and storage stages. Recent studies on freeze-dried microcapsules have pointed out that although freeze-drying is favorable for preserving heat-sensitive and water-soluble bioactives, the porous structure formed during this process may reduce the differences in final RE among different formulations. As a result, EE and RE do not always change synchronously [64]. Overall, higher SPD loading was generally beneficial for improving the EE of the microcapsules, but did not necessarily favor final retention, whereas SA played a more important auxiliary role in enhancing structural stability and improving RE.

## 4. Conclusions

In this study, SPD recovered and enriched from beech mushroom cooking liquor was successfully used to construct an SPI–Dex/SA external gelation W/O/W emulsion system and the corresponding freeze-dried microcapsules. The results showed that SPD could be effectively recovered and enriched from beech mushroom cooking liquor, and that the developed SPI–Dex/SA system provided high encapsulation efficiency, favorable structural stability, and good powder storage properties after freeze-drying. The incorporation of SA improved emulsion organization, interfacial stability, and microcapsule structure, while appropriate SPD loading contributed to enhanced encapsulation performance. Overall, the SPI–Dex/SA external gelation W/O/W emulsion and freeze-dried microcapsule system enabled effective encapsulation and stabilization of beech mushroom-derived SPD, supporting its feasibility as a delivery platform for beech mushroom-derived SPD and providing a basis for the value-added utilization of bitterness-related compounds from thermal processing by-products. However, the present study did not include direct sensory evaluation, taste-related measurements, or release characterization under simulated digestion conditions. Future work should therefore combine sensory analysis, in vitro digestion/release models, and representative food applications to verify the functional relevance of the system. In addition, the scalability and practical applicability of the process should be further evaluated by comparing freeze-drying with spray drying and by testing the compatibility of the developed system with real food processing conditions.

## Figures and Tables

**Table 1:** EE and RE of external gelation W/O/W emulsions prepared with different SPD and SA combinations.

Sample	EE (%)	RE (%)
SPD 0.1% SA 0%	92.95 ± 0.06 ^h^	93.80 ± 1.35 ^ab^
SPD 0.1% SA 0.25%	92.90 ± 0.09 ^h^	94.71 ± 0.21 ^ab^
SPD 0.1% SA 0.5%	94.28 ± 0.08 ^g^	96.02 ± 0.84 ^a^
SPD 0.1% SA 0.75%	94.37 ± 0.07 ^g^	96.87 ± 0.37 ^a^
SPD 0.1% SA 1%	95.12 ± 0.07 ^f^	96.22 ± 1.08 ^a^
SPD 0.25% SA 0%	98.10 ± 0.03 ^e^	93.17 ± 1.21 ^b^
SPD 0.25% SA 0.25%	98.37 ± 0.03 ^d^	94.46 ± 0.82 ^ab^
SPD 0.25% SA 0.5%	98.33 ± 0.03 ^d^	95.50 ± 1.13 ^ab^
SPD 0.25% SA 0.75%	98.53 ± 0.03 ^c^	96.03 ± 0.83 ^a^
SPD 0.25% SA 1%	98.51 ± 0.03 ^c^	95.24 ± 1.66 ^ab^
SPD 0.5% SA 0%	99.35 ± 0.01 ^b^	92.47 ± 0.68 ^b^
SPD 0.5% SA 0.25%	99.16 ± 0.02 ^c^	93.87 ± 0.13 ^ab^
SPD 0.5% SA 0.5%	99.43 ± 0.02 ^b^	95.11 ± 1.59 ^ab^
SPD 0.5% SA 0.75%	99.33 ± 0.01 ^b^	95.77 ± 0.85 ^a^
SPD 0.5% SA 1%	99.21 ± 0.02 ^c^	94.94 ± 0.64 ^ab^
SPD 1% SA 0%	99.68 ± 0.01 ^a^	92.75 ± 1.59 ^b^
SPD 1% SA 0.25%	99.73 ± 0.01 ^a^	92.99 ± 0.56 ^b^
SPD 1% SA 0.5%	99.70 ± 0.01 ^a^	94.41 ± 1.45 ^ab^
SPD 1% SA 0.75%	99.70 ± 0.01 ^a^	94.80 ± 0.95 ^ab^
SPD 1% SA 1%	99.76 ± 0.01 ^a^	93.97 ± 1.02 ^ab^

Data are expressed as mean ± standard deviation (n ≥ 3). Different lowercase letters in the same column indicate significant differences among samples at *p* < 0.05.

**Table 2:** EE and RE of freeze-dried microcapsules prepared with different SPD and SA combinations.

Sample	EE (%)	RE (%)
SPD 0.1% SA 0%	56.44 ± 4.79 ^g^	76.76 ± 3.41 ^c^
SPD 0.1% SA 0.25%	64.51 ± 0.72 ^f^	81.49 ± 0.86 ^b^
SPD 0.1% SA 0.5%	66.56 ± 0.68 ^f^	86.18 ± 2.14 ^a^
SPD 0.1% SA 0.75%	74.71 ± 0.65 ^de^	89.12 ± 1.26 ^a^
SPD 0.1% SA 1%	66.95 ± 0.71 ^f^	87.71 ± 1.22 ^a^
SPD 0.25% SA 0%	71.69 ± 0.63 ^e^	74.67 ± 0.82 ^cd^
SPD 0.25% SA 0.25%	73.53 ± 0.56 ^de^	79.17 ± 0.86 ^b^
SPD 0.25% SA 0.5%	87.03 ± 0.33 ^c^	84.44 ± 1.18 ^b^
SPD 0.25% SA 0.75%	73.34 ± 0.55 ^e^	87.12 ± 2.24 ^a^
SPD 0.25% SA 1%	76.96 ± 0.52 ^d^	85.52 ± 2.45 ^a^
SPD 0.5% SA 0%	86.40 ± 0.30 ^c^	71.94 ± 0.50 ^d^
SPD 0.5% SA 0.25%	93.37 ± 0.17 ^b^	76.41 ± 0.85 ^c^
SPD 0.5% SA 0.5%	94.37 ± 0.15 ^b^	82.09 ± 2.50 ^b^
SPD 0.5% SA 0.75%	96.54 ± 0.09 ^ab^	84.46 ± 2.09 ^b^
SPD 0.5% SA 1%	94.80 ± 0.13 ^ab^	82.58 ± 1.30 ^b^
SPD 1% SA 0%	97.81 ± 0.06 ^a^	70.21 ± 0.99 ^d^
SPD 1% SA 0.25%	98.13 ± 0.05 ^a^	74.09 ± 2.97 ^cd^
SPD 1% SA 0.5%	94.67 ± 0.11 ^ab^	78.60 ± 1.53 ^b^
SPD 1% SA 0.75%	97.53 ± 0.06 ^a^	81.10 ± 0.49 ^b^
SPD 1% SA 1%	96.85 ± 0.08 ^ab^	79.90 ± 1.19 ^b^

Data are expressed as mean ± standard deviation (n ≥ 3). Different lowercase letters in the same column indicate significant differences among samples at *p* < 0.05.

## Data Availability

The original contributions presented in the study are included in the article/Supplementary Materials. Further inquiries can be directed to the corresponding author.

## Supplementary Materials

The following supporting information can be downloaded at: https://www.mdpi.com/article/10.3390/foods15101734/s1, Figure S1. Creaming index of SPI–Dex/SA external gelation W/O/W emulsions loaded with different SPD levels after 7 d of storage at room temperature. Table S1. Recovery and enrichment of spermidine from cooking liquor of *Hypsizygus marmoreus* at different processing stages. Table S2. Particle size, PDI, and ζ-potential of freeze-dried microcapsules prepared with different SPD and SA combinations. Table S3. Power-law model parameters (K, n, and R²) of external gelation W/O/W emulsions prepared with different SPD and SA combinations. Table S4. Particle size and ζ-potential of freeze-dried microcapsules prepared with different SPD and SA combinations. Table S5. Water activity and moisture content of freeze-dried microcapsules prepared with different SPD and SA combinations.
