# Supplementary material for: Construction, Interfacial Characteristics, and Stability of External Gelation Soy Protein Isolate–Dextran/Sodium Alginate Water-in-Oil-in-Water Emulsions and Freeze-Dried Microcapsules Loaded with Beech Mushroom-Derived Spermidine
Source: Foods. 2026 May 14;15(10):1734. doi: 10.3390/foods15101734 (PMC13205174; doi:10.3390/foods15101734)
Supplement: Supplementary file 1 [file foods-15-01734-s001.zip › foods-4277173-supplementary.pdf]

Figure

Figure S1. Creaming index of SPI–Dex/SA external gelation W/O/W emulsions loaded with different SPD levels after 7 d of storage at room temperature.

Table

Table S1. Recovery and enrichment of spermidine from cooking liquor of *Hypsizygus marmoreus* at different processing stages
Table S2. Particle size, PDI, and ζ-potential of freeze-dried microcapsules prepared with different SPD and SA combinations
Table S3. Power-law model parameters (K, n, and R^2) of external gelation W/O/W emulsions prepared with different SPD and SA combinations
Table S4. Particle size and ζ-potential of freeze-dried microcapsules prepared with different SPD and SA combinations
Table S5. Water activity and moisture content of freeze-dried microcapsules prepared with different SPD and SA combinations

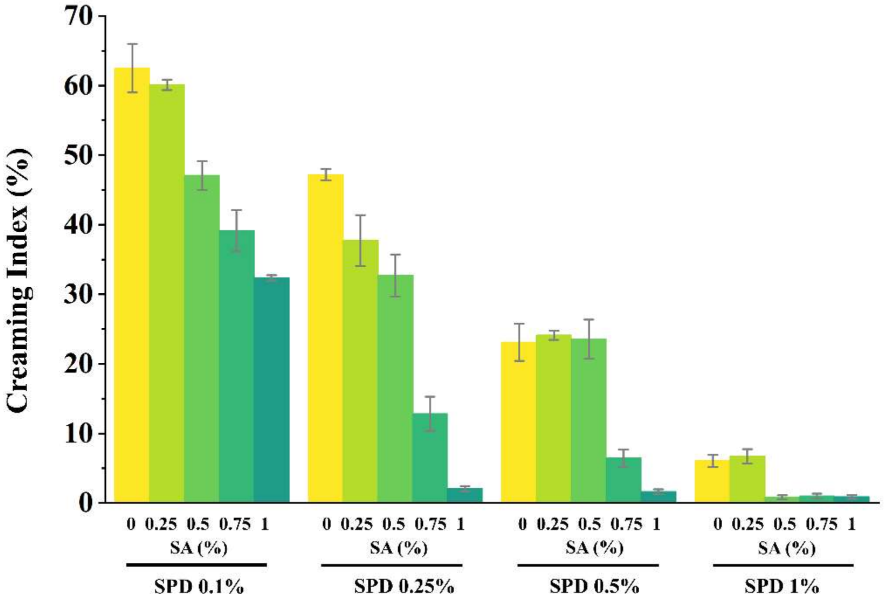

Figure S1. Creaming index of SPI–Dex/SA external gelation W/O/W emulsions loaded with different SPD levels after 7 d of storage at room temperature.

Table S1. Recovery and enrichment of spermidine from cooking liquor of *Hypsizygus marmoreus* at different processing stages

Processing stage	Volume (mL)	SPD concentration (mg/L)	Total SPD (mg)	Recovery (%)	Enrichment factor
Initial cooking liquor	500.0 ± 0.0	69.17 ± 0.00	34.59 ± 0.00	100	1
After filtration	498.0 ± 1.0	66.95 ± 1.18	33.34 ± 0.51	96.38	0.97
After concentration	50.0 ± 0.0	525.60 ± 11.84	26.28 ± 0.59	75.98	7.60
Final SPD-enriched solution	48.0 ± 0.5	520.10 ± 10.27	24.96 ± 0.49	72.16	7.52

Table S2. Particle size, PDI, and ζ-potential of external gelation W/O/W emulsions prepared with different SPD and SA combinations

Sample	Particle size (nm)	PDI	ζ-potential (mV)
SPD 0.1% SA 0%	2951.33 ± 764.76^{efg}	0.82 ± 0.11^{a}	-20.77 ± 0.76^{e}
SPD 0.1% SA 0.25%	3943.67 ± 562.31^{ef}	0.66 ± 0.11^{a}	-26.20 ± 1.15^{fghi}
SPD 0.1% SA 0.5%	3976.33 ± 546.05^{ef}	0.70 ± 0.12^{a}	-25.67 ± 1.07^{fgh}
SPD 0.1% SA 0.75%	6635.33 ± 345.17^{bc}	0.89 ± 0.10^{a}	-35.20 ± 1.42^{j}
SPD 0.1% SA 1%	7178.67 ± 878.72^{b}	0.93 ± 0.04^{a}	-16.53 ± 0.51^{cd}
SPD 0.25% SA 0%	2775.00 ± 370.96^{fg}	0.84 ± 0.13^{a}	-15.63 ± 0.65^{bc}
SPD 0.25% SA 0.25%	4284.33 ± 400.98^{de}	0.71 ± 0.11^{a}	-28.00 ± 0.52^{ghi}
SPD 0.25% SA 0.5%	5517.00 ± 310.94^{cd}	0.78 ± 0.14^{a}	-28.20 ± 1.51^{ghi}
SPD 0.25% SA 0.75%	5870.67 ± 293.67^{bc}	0.81 ± 0.12^{a}	-36.67 ± 1.85^{j}
SPD 0.25% SA 1%	9013.67 ± 593.12^{a}	0.91 ± 0.03^{a}	-25.13 ± 0.57^{fg}
SPD 0.5% SA 0%	3787.67 ± 242.31^{efg}	0.87 ± 0.11^{a}	-12.13 ± 0.55^{a}
SPD 0.5% SA 0.25%	4301.33 ± 175.00^{de}	0.89 ± 0.04^{a}	-12.90 ± 0.20^{ab}
SPD 0.5% SA 0.5%	6491.33 ± 295.47^{bc}	0.78 ± 0.15^{a}	-24.27 ± 1.01^{f}
SPD 0.5% SA 0.75%	10273.33 ± 279.70^{a}	0.84 ± 0.13^{a}	-28.67 ± 1.36^{hi}
SPD 0.5% SA 1%	3498.33 ± 514.05^{efg}	0.41 ± 0.12^{b}	-35.63 ± 1.85^{j}
SPD 1% SA 0%	3263.67 ± 394.20efg	0.71 ± 0.14^{a}	-13.67 ± 1.33^{abc}
SPD 1% SA 0.25%	3368.67 ± 67.31^{efg}	0.57 ± 0.10^{b}	-16.43 ± 0.76^{cd}
SPD 1% SA 0.5%	2482.67 ± 378.61^{g}	0.36 ± 0.03^{b}	-19.10 ± 1.08^{de}
SPD 1% SA 0.75%	3188.33 ± 130.44^{efg}	0.43 ± 0.04^{b}	-19.73 ± 0.50^{de}
SPD 1% SA 1%	6018.67 ± 676.70^{bc}	0.68 ± 0.13^{a}	-29.30 ± 1.10^{i}

Data are expressed as mean ± standard deviation ($n \geq 3$). Different lowercase letters in the same column indicate significant differences among samples ($p < 0.05$). Particle size, PDI, and ζ-potential were measured for freshly prepared emulsions after 100-fold dilution with deionized water to minimize multiple scattering effects.

Table S3. Power-law model parameters (K, n, and R^2) of external gelation W/O/W emulsions prepared with different SPD and SA combinations

Sample	K	n	R^2
SPD 0.1% SA 0%	0.0320 ± 0.0004^{e}	0.5367 ± 0.0021^{e}	0.9895 ± 0.0004^{a}
SPD 0.1% SA 0.25%	0.8062 ± 0.0097^{d}	0.6981 ± 0.0023^{c}	0.9755 ± 0.0013^{b}
SPD 0.1% SA 0.5%	3.6728 ± 0.0444^{c}	0.6759 ± 0.0023^{d}	0.9869 ± 0.0021^{a}
SPD 0.1% SA 0.75%	5.1851 ± 0.0627^{b}	0.7207 ± 0.0026^{b}	0.9892 ± 0.0005^{a}
SPD 0.1% SA 1%	11.6201 ± 0.1406^{a}	0.8150 ± 0.0023^{a}	0.9864 ± 0.0009^{a}
SPD 0.25% SA 0%	0.0738 ± 0.0012^{e}	0.5144 ± 0.0126^{d}	0.9817 ± 0.0037^{b}
SPD 0.25% SA 0.25%	1.9800 ± 0.0429^{d}	0.5297 ± 0.0084^{d}	0.9966 ± 0.0006^{a}
SPD 0.25% SA 0.5%	4.1052 ± 0.0496^{c}	0.6831 ± 0.0078^{c}	0.9814 ± 0.0019^{b}
SPD 0.25% SA 0.75%	5.1260 ± 0.0857^{b}	0.7572 ± 0.0065^{b}	0.9921 ± 0.0018^{ac}
SPD 0.25% SA 1%	10.0030 ± 0.1338^{a}	0.7993 ± 0.0097^{a}	0.9837 ± 0.0068^{bc}
SPD 0.5% SA 0%	0.0861 ± 0.0046^{e}	0.7256 ± 0.0025^{a}	0.9733 ± 0.0016^{b}
SPD 0.5% SA 0.25%	0.4903 ± 0.0332^{d}	0.4557 ± 0.0051^{c}	0.9790 ± 0.0009^{bc}
SPD 0.5% SA 0.5%	1.1076 ± 0.0198^{c}	0.4297 ± 0.0029^{d}	0.9869 ± 0.0035^{ac}
SPD 0.5% SA 0.75%	1.7996 ± 0.0293^{b}	0.4170 ± 0.0067^{d}	0.9895 ± 0.0047^{a}
SPD 0.5% SA 1%	4.3661 ± 0.0219^{a}	0.4861 ± 0.0084^{b}	0.9927 ± 0.0034^{a}
SPD 1.0% SA 0%	0.0216 ± 0.0002^{e}	0.7728 ± 0.0032^{a}	0.9901 ± 0.0029^{a}
SPD 1.0% SA 0.25%	2.4284 ± 0.0280^{d}	0.5303 ± 0.0027^{e}	0.9944 ± 0.0034^{a}
SPD 1.0% SA 0.5%	7.0815 ± 0.0684^{c}	0.5460 ± 0.0049^{d}	0.9943 ± 0.0028^{a}
SPD 1.0% SA 0.75%	10.7881 ± 0.1483^{b}	0.5730 ± 0.0023^{c}	0.9920 ± 0.0031^{a}
SPD 1.0% SA 1%	15.4875 ± 0.1223^{a}	0.6694 ± 0.0033^{b}	0.9942 ± 0.0026^{a}

Data are expressed as mean ± standard deviation (n = 3). Different lowercase letters within the same SPD level indicate significant differences among samples with different SA concentrations ($p < 0.05$).

Table S4. Particle size, PDI, and ζ-potential of freeze-dried microcapsules prepared with different SPD and SA combinations

Sample	Particle size (nm)	PDI	ζ-potential (mV)
SPD 0.1% SA 0%	5080.00 ± 646.99^{f}	0.31 ± 0.07^{abc}	-26.03 ± 0.31^{e}
SPD 0.1% SA 0.25%	5378.00 ± 276.18^{ef}	0.27 ± 0.04^{abc}	-28.07 ± 0.32^{f}
SPD 0.1% SA 0.5%	$5879.33 \pm 488.03^{cdef}$	0.24 ± 0.04^{abc}	-37.03 ± 0.67^{i}
SPD 0.1% SA 0.75%	7157.33 ± 263.03^{abc}	0.22 ± 0.07^{abc}	-36.30 ± 0.36^{hi}
SPD 0.1% SA 1%	7591.67 ± 280.04^{ab}	0.20 ± 0.03^{b}	-44.30 ± 0.87^{j}
SPD 0.25% SA 0%	5501.33 ± 389.26^{def}	0.39 ± 0.10^{abc}	-21.03 ± 0.49^{abc}
SPD 0.25% SA 0.25%	7152.33 ± 235.70^{abc}	0.34 ± 0.04^{abc}	-26.10 ± 0.46^{e}
SPD 0.25% SA 0.5%	7717.67 ± 954.22^{ab}	0.32 ± 0.09^{abc}	-27.83 ± 0.38^{ef}
SPD 0.25% SA 0.75%	$5868.00 \pm 479.23^{cdef}$	0.27 ± 0.08^{abc}	-31.23 ± 0.49^{g}
SPD 0.25% SA 1%	$6161.33 \pm 287.27^{bcdef}$	0.22 ± 0.04^{bc}	-35.03 ± 0.76^{h}
SPD 0.5% SA 0%	$7050.67 \pm 540.23^{abcd}$	0.45 ± 0.11^{a}	-20.90 ± 0.36^{ab}
SPD 0.5% SA 0.25%	8081.67 ± 842.20^{a}	0.41 ± 0.09^{abc}	-22.47 ± 0.21^{bcd}
SPD 0.5% SA 0.5%	7208.00 ± 546.79^{abc}	0.33 ± 0.05^{abc}	-28.33 ± 1.07^{f}
SPD 0.5% SA 0.75%	7179.00 ± 312.35^{abc}	0.29 ± 0.07^{abc}	-30.40 ± 0.46^{g}
SPD 0.5% SA 1%	$6321.33 \pm 230.20^{bcdef}$	0.21 ± 0.04^{b}	-34.50 ± 0.35^{h}
SPD 1% SA 0%	$6632.67 \pm 273.92^{abcdef}$	0.37 ± 0.09^{abc}	-23.77 ± 0.70^{d}
SPD 1% SA 0.25%	$6452.67 \pm 95.56^{bcdef}$	0.44 ± 0.12^{ac}	-19.63 ± 0.67^{a}
SPD 1% SA 0.5%	7227.00 ± 513.89^{abc}	0.40 ± 0.09^{abc}	-20.93 ± 0.31^{ab}
SPD 1% SA 0.75%	$6942.67 \pm 784.04^{abcde}$	0.34 ± 0.07^{abc}	-27.00 ± 1.05^{ef}
SPD 1% SA 1%	8077.00 ± 685.63^{a}	0.38 ± 0.09^{abc}	-22.90 ± 0.82^{cd}

Data are expressed as mean ± standard deviation ($n \geq 3$). Different lowercase letters in the same column indicate significant differences among samples ($p < 0.05$).

Table S5. Water activity and moisture content of freeze-dried microcapsules prepared with different SPD and SA combinations

Sample	Water activity (aw)	Moisture content (%)
SPD 0.1% SA 0%	0.2194 ± 0.0038^{cd}	2.41 ± 0.11^{de}
SPD 0.1% SA 0.25%	0.2248 ± 0.0046^{ab}	2.86 ± 0.14^{ab}
SPD 0.1% SA 0.5%	0.2227 ± 0.0041^{bc}	2.31 ± 0.09^{ef}
SPD 0.1% SA 0.75%	0.2163 ± 0.0035^{ef}	2.22 ± 0.10^{fg}
SPD 0.1% SA 1%	0.2176 ± 0.0042^{de}	2.48 ± 0.12^{d}
SPD 0.25% SA 0%	0.2187 ± 0.0039^{de}	2.29 ± 0.08^{ef}
SPD 0.25% SA 0.25%	0.2158 ± 0.0044^{f}	2.64 ± 0.13^{c}
SPD 0.25% SA 0.5%	0.2139 ± 0.0036^{g}	2.14 ± 0.09^{g}
SPD 0.25% SA 0.75%	0.2279 ± 0.0051^{a}	2.71 ± 0.15^{bc}
SPD 0.25% SA 1%	0.2231 ± 0.0047^{bc}	2.79 ± 0.12^{ab}
SPD 0.5% SA 0%	0.2241 ± 0.0043^{ab}	2.53 ± 0.10^{cd}
SPD 0.5% SA 0.25%	0.2212 ± 0.0037^{c}	2.36 ± 0.11^{e}
SPD 0.5% SA 0.5%	0.2224 ± 0.0045^{bc}	2.44 ± 0.09^{de}
SPD 0.5% SA 0.75%	0.2268 ± 0.0049^{a}	2.68 ± 0.14^{bc}
SPD 0.5% SA 1%	0.2236 ± 0.0040^{bc}	2.18 ± 0.08^{g}
SPD 1% SA 0%	0.2229 ± 0.0038^{bc}	2.27 ± 0.09^{ef}
SPD 1% SA 0.25%	0.2243 ± 0.0044^{ab}	2.55 ± 0.10^{cd}
SPD 1% SA 0.5%	0.2235 ± 0.0041^{bc}	2.61 ± 0.11^{c}
SPD 1% SA 0.75%	0.2201 ± 0.0048^{cd}	2.88 ± 0.16^{a}
SPD 1% SA 1%	0.2217 ± 0.0039^{c}	2.63 ± 0.13^{c}

Measurements were performed on the same batch of freeze-dried microcapsules obtained from three independent freeze-drying replicates. Values are expressed as mean ± standard deviation ($n = 3$). Different lowercase letters indicate significant differences ($p < 0.05$).
